# Advantages of Widefield Optical Coherence Tomography in the Diagnosis of Retinopathy of Prematurity

**DOI:** 10.3389/fped.2021.797684

**Published:** 2022-01-18

**Authors:** Thanh-Tin P. Nguyen, Shuibin Ni, Shanjida Khan, Xiang Wei, Susan Ostmo, Michael F. Chiang, Yali Jia, David Huang, Yifan Jian, J. Peter Campbell

**Affiliations:** ^1^School of Medicine, Casey Eye Institute, Oregon Health and Science University, Portland, OR, United States; ^2^Department of Biomedical Engineering, Casey Eye Institute, Oregon Health and Science University, Portland, OR, United States; ^3^National Eye Institute, National Institutes of Health, Bethesda, MD, United States

**Keywords:** retinopathy of prematurity, pediatric retina, optical coherence tomography, handheld optical coherence tomography, optical coherence tomography with angiography

## Abstract

Recent advances in portable optical coherence tomography (OCT) and OCT angiography (OCTA) have resulted in wider fields of view (FOV) and shorter capture times, further expanding the potential clinical role of OCT technology in the diagnosis and management of retinopathy of prematurity (ROP). Using a prototype, handheld OCT device, retinal imaging was obtained in non-sedated infants in the neonatal intensive care unit (NICU) as well as sedated infants in the operating room of Oregon Health & Science University (OHSU) Hospital. In this observational study, we provide an overview of potential advantages of OCT-based disease assessment in ROP. We observed that next-generation OCT imaging (a) may be sufficient for objective diagnosis and zone/stage/plus disease categorization, (b) allows for minimally-invasive longitudinal monitoring of disease progression and post-treatment course, (c) provides three-dimensional mapping of the vitreoretinal interface, and (d) with OCTA, enables dye-free visualization of normal and pathologic vascular development.

## Introduction

Optical coherence tomography (OCT) and OCT angiography (OCTA) are widely used to evaluate patients with diseases of the macula and vitreoretinal interface. Over the last two decades, the diagnosis and management of both leading causes of blindness in adults (diabetic retinopathy and age-related macular degeneration) have incorporated OCT into the standard of care. Applications in pediatric retinal diseases, such as retinopathy of prematurity (ROP), have been more challenging due to the difficulty of imaging children using devices designed for adults, both in terms of positioning requirements of adult table-mounted devices as well as the need for patient cooperation to prevent motion artifacts. Recent advancements in OCT technology have made image acquisition feasible for use in awake infants and have the potential to improve our management of many diseases in pediatric retina, including ROP, by allowing for objective diagnosis and sensitive detection of anatomical changes.

Previous ROP studies incorporating OCT technology have largely directed attention at the diagnostic value of macular and posterior retinal changes due to practical FOV limitations. These efforts have broadly fallen into two categories: detection of subclinical pathology and evaluating objective biomarkers of disease. We have learned that OCT can demonstrate retinoschisis (1) and early vitreoretinal traction (2) better than our eyes can appreciate during the clinical examination, that cystoid macular changes can occur in ROP (3, 4) as in other retinal vascular diseases, and that the changes associated with plus disease cause three-dimensional architectural changes in the retina (5), including at the vascular-avascular border (6, 7). There has also been increased attention on the sequelae of blood retinal barrier disruption, with an increase in vitreous opacities seen in more severe ROP (8). Recent papers have focused on normal and abnormal foveal development in prematurity (9, 10), previously impossible to evaluate *in vivo*, as well as the repeatability and reproducibility of measurements taken with a handheld OCT device (11).

Over the last 10 years there has been a transition toward faster laser speeds using swept-source (SS-OCT) designs in handheld OCT prototypes, which has enabled faster image acquisition and OCTA (12–14). With OCT in general, there is a tradeoff between signal quality, imaging speed, and FOV. In the pediatric population, since imaging non-sedated infants necessitates fast imaging speed to prevent motion artifacts, the tradeoff is between signal quality and FOV. For OCTA, this reality means that given current imaging speeds, OCTA can be obtained at small FOVs but motion artifact becomes significant as FOV is expanded. Image segmentation also remains a challenging barrier, especially given motion artifact, and has thus far prevented validation of quantitative OCTA biomarkers in neonates.

We previously described a widefield, handheld SS-OCT device with a >55° FOV, that was capable of visualizing peripheral pathology when coupled with scleral depression (15). Subsequently, we have re-engineered the FOV to approximately 105° in a new design, which can provide real-time *en face* visualization that can be used to optimize the image quality and orientation prior to image acquisition, which takes 1.5 s. (16) Our experience operating this device in the NICU has provided a number of observations as to how advances in OCT technology may provide value to patient care in the future. In this paper, we review the potential advantages of using widefield OCT in the diagnosis and management of ROP.

## Methods

**Figure 1:**
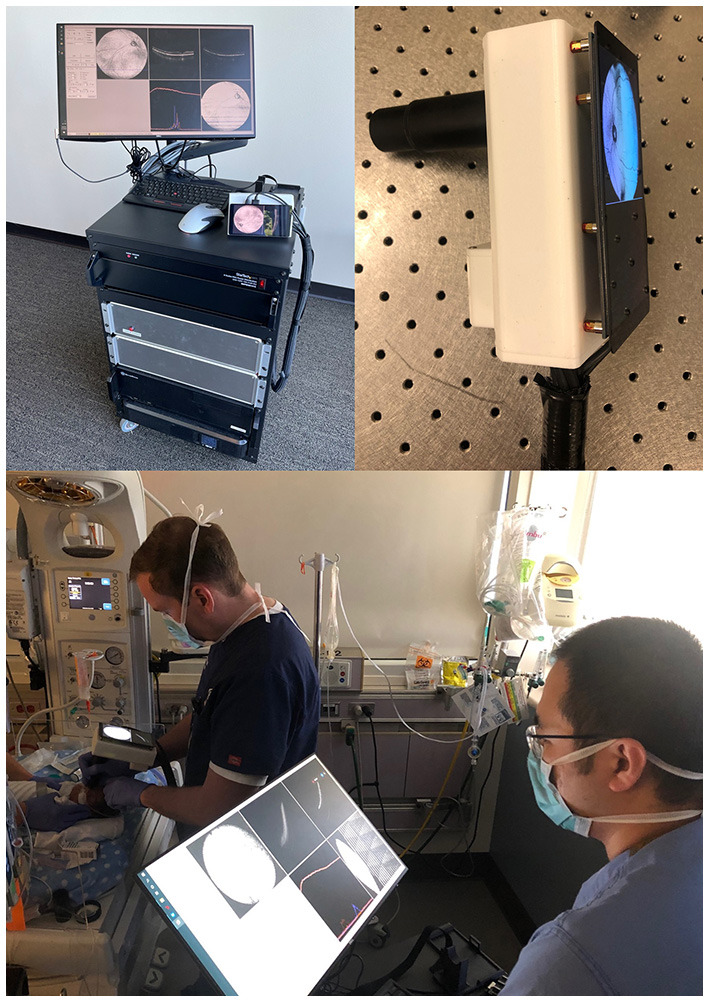
Portable handheld OCT device. Upper-left image shows the prototype device with OCT module and computer. Upper-right image shows a close-up of the probe with a 5.5-inch display capable of real-time *en face* visualization. Bottom image shows the imaging process of an infant in the neonatal intensive care unit.

This study was approved by the Institutional Review Board (IRB) at Oregon Health & Science University (OHSU) and adheres to all tenets of the Declaration of Helsinki. Infants were eligible for recruitment if they met criteria for ROP screening (birthweight ≤ 1,500 g or gestational age ≤ 30 weeks). Exams were performed at the bedside with an eyelid speculum, after administration of cyclomydril and proparacaine. Consent for imaging was obtained from parents. Between March and November 2021, we performed more than 200 eye examinations with OCT. Infants were imaged with a 400-kHz portable handheld SS-OCT system with a modular lens system as shown in Figure 1, providing up to a 105° FOV. The camera was held by the examining ophthalmologist and a second person controls the software.

Our investigational system features real-time *en face* visualization to allow the operator visual feedback of the scanning region. OCT volumes were averaged in linear scale, and processed using custom software to create mean-intensity *en face* projections. B-scans were processed using an image registration algorithm. This enables the OCT to be used as a fundus camera for visualization of peripheral ROP stage even prior to acquisition of the OCT volumes. Image acquisition takes 1.5 s.

In post-processing, three-dimensional rendering was completed in the Volume Viewer plugin of Fiji, a distribution of ImageJ (17). Segmentation of OCT volumes was performed manually with the Insight Toolkit (18), then interpolated and applied to volumes using custom software coded in MATLAB (19). OCTA images were generated using a novel phase-stabilized complex-decorrelation methodology (20), with automated segmentation performed using a guided bidirectional graph search method (21), both of which were designed specifically for use in swept-source, widefield applications.

## Results

### Objective Diagnosis/Documentation

**Figure 2:**
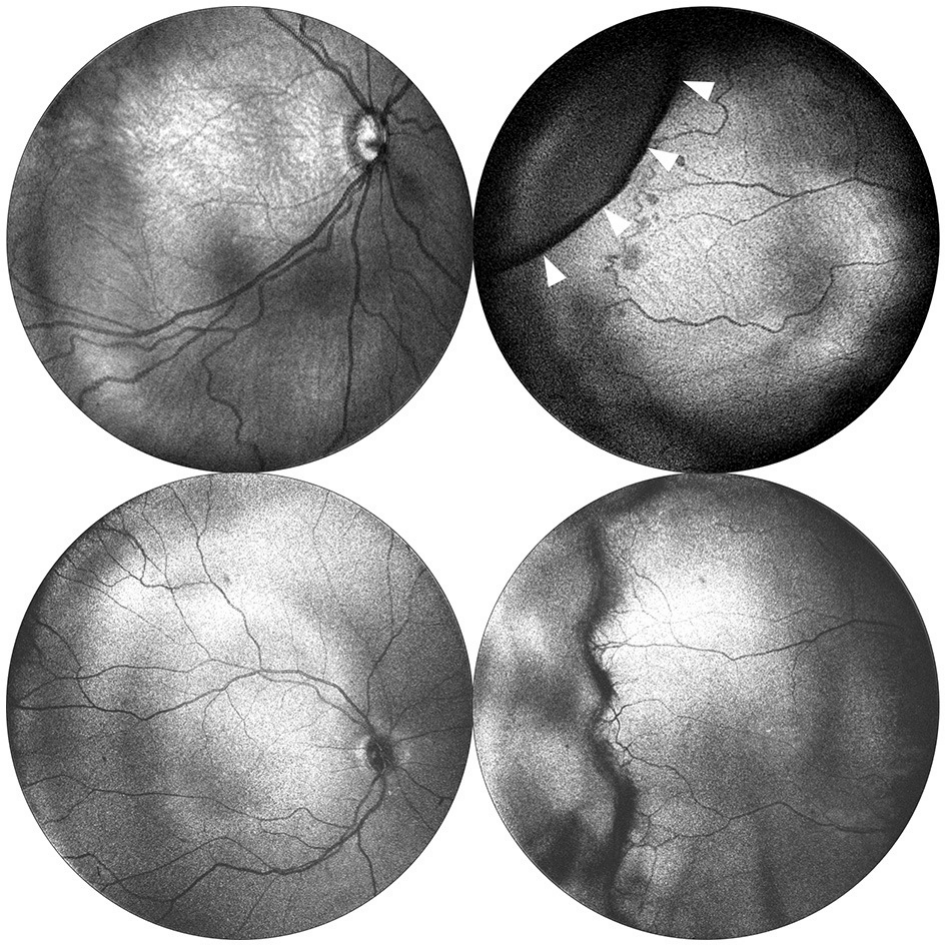
Posterior and peripheral *en face* images obtained via portable 55° and 105° FOV OCT. Images in the top row were obtained using a 55° FOV system from a baby born at 24 weeks gestation (770 grams) and imaged at 33 weeks postmenstrual age. The images in the bottom row were obtained using a 105° FOV system from a baby born at 30 weeks gestation (1,390 g), and imaged at 40 weeks postmenstrual age. Posterior images are shown in the left column and demonstrate the expanded FOV. Peripheral images are shown in the right column and were obtained with the aid of scleral depression using the 55° system. White arrows indicate the indentation of the scleral depressor.

We found that real-time *en face* visualization allows handheld OCT to be used much like an ophthalmoscope, with a comparable FOV and improved contrast when visualizing the border between vascular and avascular retina. Since visualization of peripheral stage typically requires scleral depression, we evaluated whether scleral depression, coupled with the handheld OCT could detect and visualize peripheral stage. Figure 2 demonstrates posterior and peripheral images obtained using this method, using both 55° and 105° FOV devices. In most cases, it was possible to objectively assess the degree of peripheral stage. The cases where we were unable to visualize the retinal periphery using OCT were also the most challenging to examine ophthalmoscopically. These included babies with swollen eyelids due to continuous positive airway pressure (CPAP), and those who were unstable clinically, where we prioritized the clinical standard ophthalmoscopic examination to rule out clinically significant disease but otherwise kept the examination as brief as possible to minimize any added stress and risk from research imaging. That said, in those most challenging cases where we were able to obtain OCT imaging, we often found that *en face* OCT provided better sensitivity for detection of subtle changes at the vascular-avascular border, although this was not performed in a masked fashion.

### Monitoring of Disease Progression and Regression

**Figure 3:**
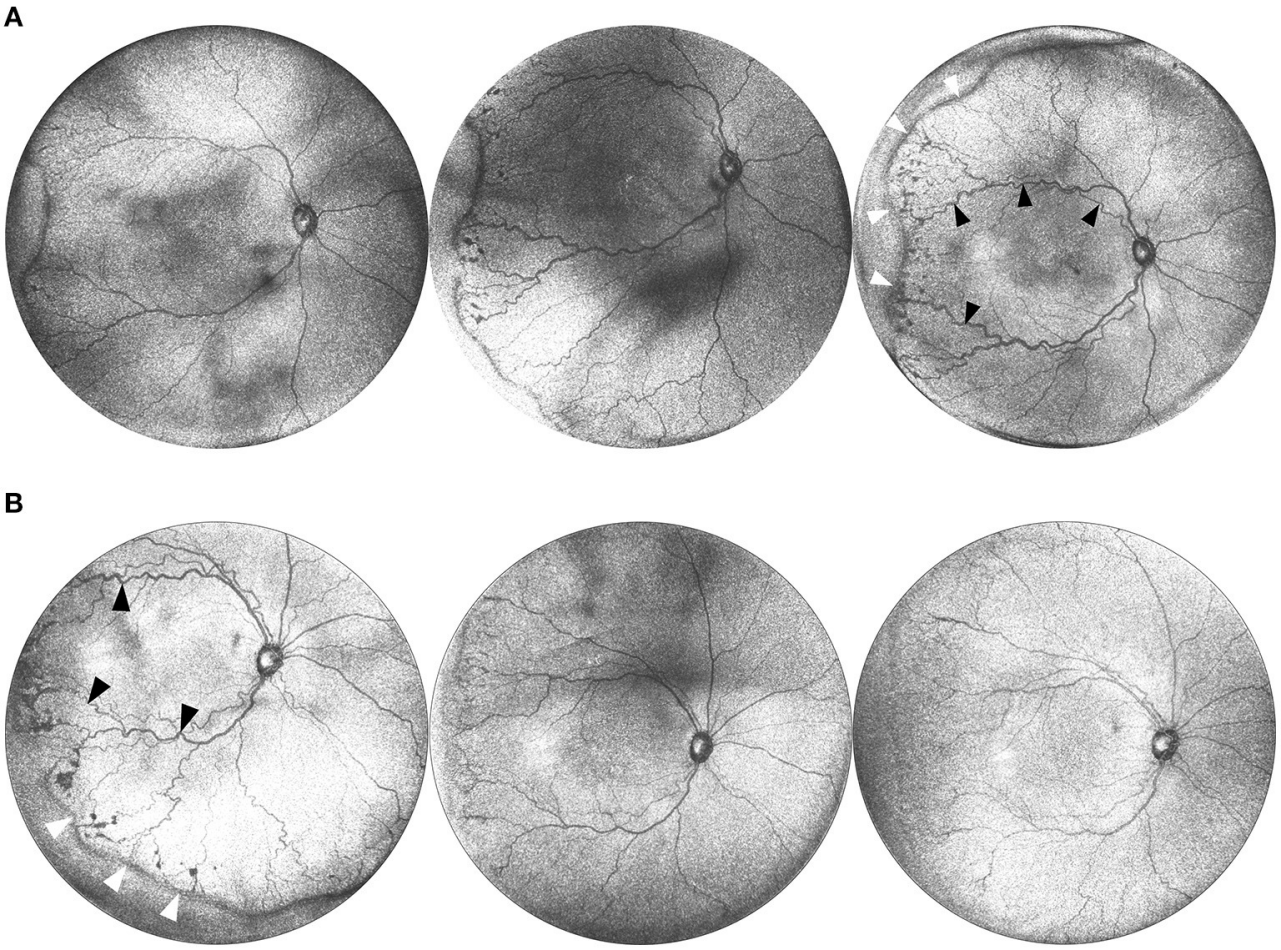
**(A)** Serial widefield OCT images taken on three successive weeks. These images are from a baby born at 25 weeks gestation (449 g) and imaged at 36, 37, and 38 weeks postmenstrual age. Serial imaging shows the posterior and peripheral retina in one image, and tracks the progression of arterial and venous tortuosity and fibrovascular proliferation. Black arrows highlight areas of increased dilation and tortuosity. White arrows indicate the peripheral ridge which has increased over the time period, along with popcorn neovascularization posterior to the ridge. **(B)** Serial images pre and post treatment. This is the same infant from **(A)** at postmenstrual age 38, 39, and 40 weeks. The leftmost image shows a posterior *en face* view taken immediately prior to treatment with 0.625 mg intravitreal bevacizumab. Middle image shows regression of disease 1 week after treatment, and rightmost image shows further regression 2 weeks after treatment. Black arrows indicate areas of vascular tortuosity pre-treatment that improve post-treatment. White arrows indicate areas of pathologic neovascularization that appears less dense post-treatment.

Serial evaluation of fundus photos has demonstrated value in detecting signs of disease progression (22). We found that *en face* visualization provided sufficient detail to assess relative changes in disease severity across time (Figure 3A) by direct comparison to prior images. Additionally, when evaluating the effect of treatment with intravitreal bevacizumab, we found that direct comparison of the *en face* OCT of the posterior pole and retinal periphery demonstrated reduction in the stage and extent of peripheral disease as well as the degree of plus disease (Figure 3B).

### Vitreoretinal Interface and Retinal Detachment

**Figure 4:**
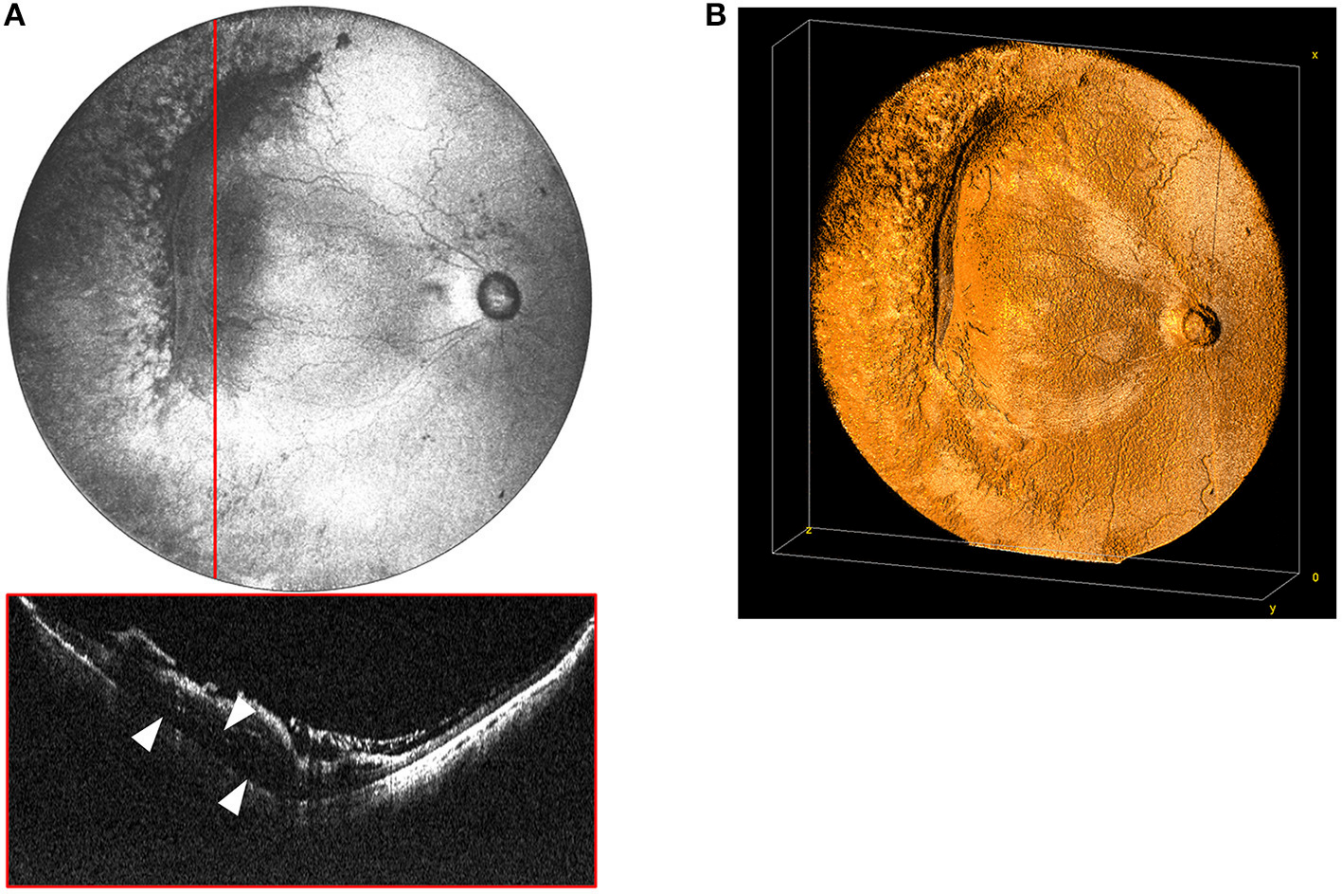
**(A)** Widefield *en face* OCT image of a stage 4A tractional retinal detachment secondary to ROP. These images are from a baby born at 25 weeks gestation (571 g) and imaged at 50 weeks postmenstrual age. Red horizontal line indicates location of the corresponding cross-sectional B-scan. White arrows designate area of retinal detachment with subretinal fluid. **(B)** Volumetric rendering of retinal detachment shown in 4A. This visualization enables topographic representation of the three-dimensional relationships in severe retinopathy of prematurity, including highlighting the elevation of the blood vessels and fibrovascular proliferation causing the retinal detachment.

A major advantage of OCT over the ophthalmoscopic examination in vitreoretinal disease management is the ability to determine three-dimensional relationships between the vitreous, the retina, and extraretinal membranes. While these relationships are visible with smaller FOV systems, it takes far more time to visualize the entire periphery with multiple scans. With the 105° FOV system, we can capture both the posterior pole and retinal mid-periphery in a single image, allowing for three-dimensional reconstruction of the topographical anatomy of the retina and vitreous. Figure 4A demonstrates how these three-dimensional relationships can be visualized in the setting of a retinal detachment using cross-sectional B-scans. Additionally, three-dimensional rendering provides another means of interactive visualization, which may aid surgical planning (Figure 4B).

**Figure 5:**
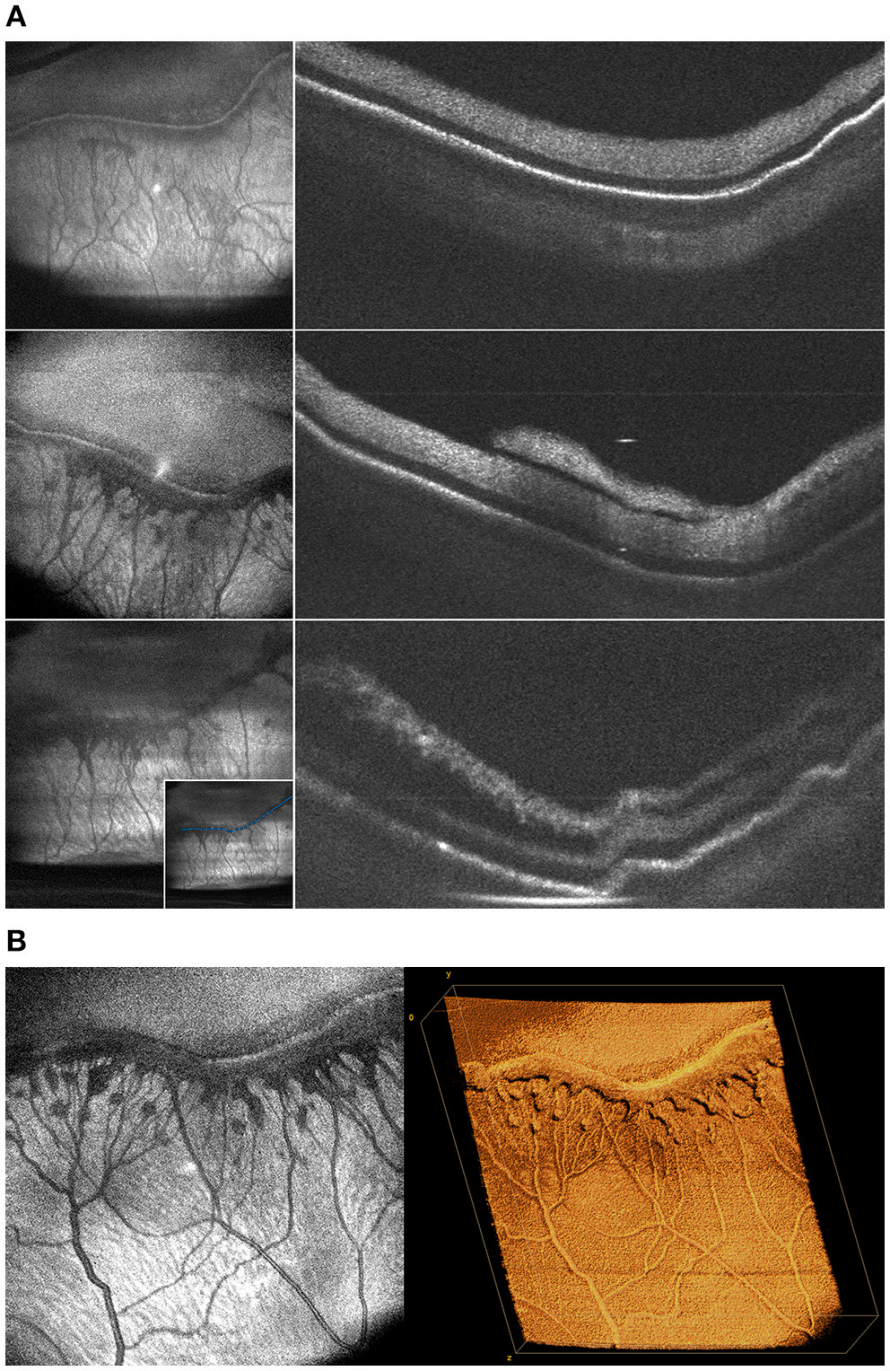
**(A)** Changes in the morphology of fibrovascular proliferation demonstrated by cross-sectional B-scans taken along the length of the ridgeline every 2 weeks. These images were obtained from a baby born 25 weeks gestation and seen at 36, 39, and 41 weeks postmenstrual age. Left column shows *en face* projection of peripheral pathology. Right column shows progression of neovascularization and retinal traction on B-scan. The inset in the bottom-left image shows sample manual tracing of the ridgeline B-scan used to generate these cross-sections. **(B)** Volumetric rendering of ridgeline in stage 3 ROP. This is the same patient as **(A)** seen at 39 weeks. The image on the left shows an *en face* view of peripheral fibrovascular proliferation in stage 3 ROP. The image on the right shows three-dimensional rendering of the same volume.

Using the 55° and 105° FOV systems, we imaged patients with a broad range of peripheral stage, as shown in Figures 3, 5. Note that eyes can have differences both in the degree and extent of peripheral stage as well as the degree of dilation and tortuosity of the posterior retinal vessels. B-scans drawn along the length of the vascular-avascular junction demonstrate three-dimensional retinal changes (Figure 5A) and could one day be a more precise and continuous biomarker for disease severity in an eye than “maximum stage,” which the current classification system recommends. OCT also aids in the diagnosis of popcorn neovascularization, considered in the spectrum of stage 2 ROP, and can show how extraretinal fibrovascular proliferation coalesces into the typical stage 3 lesion appearance (Figures 3, 5).

### Optical Coherence Tomography Angiography

**Figure 6:**
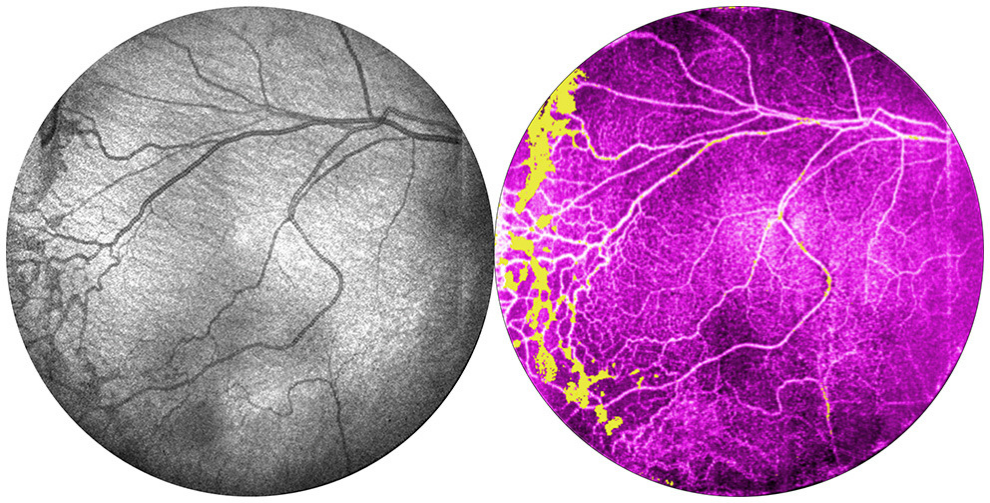
Comparison of OCT and OCTA images of retinal vasculature. This is the same patient as Figure 1 (24 weeks gestation, 770 g) seen at 35 weeks gestation. From left: OCT *en face* image, and corresponding OCTA *en face* image with flow signal in the vitreous shown in yellow.

Figure 6 shows OCTA *en face* projections obtained with the widefield OCT device. With OCTA, there is a tradeoff between FOV and motion artifact as seen in the example. In the future, this may be reduced by using a smaller FOV, with a faster laser, or with improved software. Nonetheless, these results demonstrate the potential for non-invasive angiographic visualization of non-perfusion and neovascularization, both of which may be quantifiable biomarkers in the future.

## Discussion

Advances in the speed and optical engineering of OCT devices have made the incorporation of OCT into ROP diagnosis more practical. In this paper, we describe key potential advantages of widefield OCT in the diagnosis and management of ROP.

Widefield imaging using OCT has the potential to provide quantitative and objective assessment of each of the components of ROP classification. The diagnosis of ROP has always been subjective and qualitative, with the clinician responsible for determining the zone, stage, and presence of plus disease. Yet each of these components represents an ordinal categorization of a continuous spectrum that could be measured more discretely using OCT. Zone represents the area of vascularized retina, which could in theory be measured as an area using *en face* OCT given the high contrast possible on OCT images. As shown in Figure 3A, stage represents a continuous spectrum that slowly evolves over time until it is eventually treated, or spontaneously regresses (23). In the future, it may be feasible to automatically segment the peripheral vascular-avascular junction and quantify thickness to create objective cutoffs for stage that correlate with population-derived treatment thresholds. Finally, the spectrum of plus disease has been well-described, and may be more objectively quantified using artificial intelligence-derived metrics (24).

Previous work has demonstrated that there is a relationship between plus disease severity on a posterior pole photograph, the zone of disease, and the degree and number of clock hours of peripheral stage in an eye on ophthalmoscopy (24). Widefield OCT has the potential to directly visualize these relationships and provide rapid, non-invasive assessment of ROP severity. Each of these biomarkers may be followed over time to directly assess disease progression (25) or regression (23). This has implications not only for improved clinical care, but also for research as it has not previously been possible to directly measure the continued growth of the retinal vasculature *in vivo*, and the changes associated with the development of pathologic neovascularization. The natural course and response to treatment of ROP can be followed longitudinally with more frequent imaging, which may allow for earlier detection of changes and correspondingly reduce the time to treatment.

The advantage of widefield OCT over fundus photography is even greater for eyes with moderate or severe ROP where the topographic changes in the vitreoretinal interface are more dramatic. In our experience, these changes are easier to appreciate using OCT, especially as stage 2 with popcorn neovascularization develops. These lesions often coalesce into typical stage 3 appearance (as in Figures 3, 5). Technically, the distinction between stage 2 and stage 3 is when “extraretinal” neovascularization develops, but practically, the diagnosis is clinical and based on the ophthalmoscopic appearance, since popcorn neovascularization represents extraretinal neovascularization. This is a great example of how OCT and OCTA could lead to more precise classifications of ROP stage in the future, since the transition when neovascularization breaks through the inner limiting membrane (ILM), which can be observed *ex vivo* on histology (6), but not *in vivo*, is a critical turning point in the risk of retinal detachment and blindness. Previous work has highlighted the significance of vitreous opacities, which are presumably signs of exudation from breakdown of the blood-retinal barrier secondary to vascular endothelial cell dysfunction (8). Being able to directly evaluate the three-dimensional anatomy of the peripheral retina, as well as the vitreous, may help determine whether these findings are simply a surrogate marker of ROP severity, or independent prognostically. Finally, the use of OCT for early detection and diagnosis of retinal detachment could dramatically reduce the likelihood of blindness. Since OCT is far more sensitive for the detection of early vitreoretinal traction, it is hard to imagine that with appropriate follow-up, extensive stage 3 and early stage 4 ROP would be missed.

As OCTA technology advances, additional biomarkers relating to avascular zones or neovascular flow may be discovered and incorporated into clinical care. Fluorescein angiography (FA) has been shown to increase sensitivity of ROP screening and improve inter-observer agreement regarding classification (26). OCTA provides an alternative to FA that does not require intravenous contrast. Perhaps the most significant impact of widefield OCT in the evaluation of premature babies is what we have yet to discover. In adults, OCT is increasingly being used to predict the presence of systemic disease, in particular neurological and cardiovascular disease. ROP exists in a larger collection of comorbid diseases with similar etiology, with hyperoxia induced vascular injury in capillary beds throughout the body. Clinically, this is most relevant in the brain, lung, gastrointestinal tract, and kidneys, and it may be that microvascular or structural alterations in the eye, detectable with OCT or OCTA, indicate the presence of comorbid brain or lung disease. Indeed, there is already some indication that this is the case, with recent work demonstrating abnormalities in retinal thickness associated with birthweight, and prior history of sepsis and necrotizing enterocolitis (27).

## Conclusion

In this paper, we have attempted to argue for the advantages of widefield OCT in the diagnosis and management of ROP. The main limitation of this work is that there are currently no commercially available devices that allow clinicians to incorporate these findings in clinical care, or develop further evidence for the role of OCT in the management of ROP. As this technology continues to improve, and costs come down, we believe that it is a matter of time before faster, higher resolution, and low-artifact OCTA may produce even more useful biomarkers for *in vivo* assessment of disease. We hope that this work may stimulate others to innovate further and for the added value of this technology to be clearly seen such that commercially available devices may be made available to improve the care of infants at risk for ROP around the world.

## Data Availability Statement

The original contributions presented in the study are included in the article/supplementary material, further inquiries can be directed to the corresponding author/s.

## Ethics Statement

The studies involving human participants were reviewed and approved by Oregon Health and Science University Institutional Review Board. Written informed consent to participate in this study was provided by the participants' legal guardian/next of kin. Written informed consent was obtained from the minor(s)' legal guardian/next of kin for the publication of any potentially identifiable images or data included in this article.

## Author Contributions

JC, MC, DH, YaJ, and YiJ designed the study and obtained the funding. SO developed and maintained the patient database and consented all patients. T-TN, SN, SK, and XW identified, processed, and contributed images to the final paper. All authors provided critical review and approved the final version of the manuscript.

## Funding

This work was supported by grants R01 HD107494 and P30 EY10572 from the National Institutes of Health (Bethesda, MD), by unrestricted departmental funding, a Career Development Award (JC) and a Career Advancement Award (YiJ) from Research to Prevent Blindness (New York, NY), and the West Coast Consortium for Technology and Innovations in Pediatrics. The sponsors or funding organizations had no role in the design or conduct of this research.

## Conflict of Interest

Oregon Health & Science University (OHSU), DH, and YaJ have significant financial interests in Optovue, a company that may have a commercial interest in the results of this research and technology. These potential conflicts of interest have been reviewed and managed by OHSU. DH and YaJ have received royalties for patent files through OHSU, as well as loaned equipment for research from Optovue. The remaining authors declare that the research was conducted in the absence of any commercial or financial relationships that could be construed as a potential conflict of interest.

## Publisher's Note

All claims expressed in this article are solely those of the authors and do not necessarily represent those of their affiliated organizations, or those of the publisher, the editors and the reviewers. Any product that may be evaluated in this article, or claim that may be made by its manufacturer, is not guaranteed or endorsed by the publisher.
